# Distinct molecular subtypes of systemic sclerosis and gene signature with diagnostic capability

**DOI:** 10.3389/fimmu.2023.1257802

**Published:** 2023-10-02

**Authors:** Qi Wang, Chen-Long Li, Li Wu, Jing-Yi Hu, Qi Yu, Sheng-Xiao Zhang, Pei-Feng He

**Affiliations:** ^1^ School of Basic Medical Sciences, Shanxi Medical University, Taiyuan, China; ^2^ Shanxi Key Laboratory of Big Data for Clinical Decision Research, Taiyuan, China; ^3^ Department of Anesthesiology , Shanxi Provincial People’s Hospital (Fifth Hospital) of Shanxi Medical University, Taiyuan, China; ^4^ School of Management, Shanxi Medical University, Taiyuan, China; ^5^ Department of Rheumatology, Second Hospital of Shanxi Medical University, Taiyuan, China

**Keywords:** systemic sclerosis, unsupervised machine learning, molecular subtypes, immune microenvironment, diagnostic

## Abstract

**Background:**

As Systemic Sclerosis (SSc) is a connective tissue ailment that impacts various bodily systems. The study aims to clarify the molecular subtypes of SSc, with the ultimate objective of establishing a diagnostic model that can inform clinical treatment decisions.

**Methods:**

Five microarray datasets of SSc were retrieved from the GEO database. To eliminate batch effects, the combat algorithm was applied. Immune cell infiltration was evaluated using the xCell algorithm. The ConsensusClusterPlus algorithm was utilized to identify SSc subtypes. Limma was used to determine differential expression genes (DEGs). GSEA was used to determine pathway enrichment. A support vector machine (SVM), Random Forest(RF), Boruta and LASSO algorithm have been used to select the feature gene. Diagnostic models were developed using SVM, RF, and Logistic Regression (LR). A ROC curve was used to evaluate the performance of the model. The compound-gene relationship was obtained from the Comparative Toxicogenomics Database (CTD).

**Results:**

The identification of three immune subtypes in SSc samples was based on the expression profiles of immune cells. The utilization of 19 key intersectional DEGs among subtypes facilitated the classification of SSc patients into three robust subtypes (gene_ClusterA-C). Gene_ClusterA exhibited significant enrichment of B cells, while gene_ClusterC showed significant enrichment of monocytes. Moderate activation of various immune cells was observed in gene_ClusterB. We identified 8 feature genes. The SVM model demonstrating superior diagnostic performance. Furthermore, correlation analysis revealed a robust association between the feature genes and immune cells. Eight pertinent compounds, namely methotrexate, resveratrol, paclitaxel, trichloroethylene, formaldehyde, silicon dioxide, benzene, and tetrachloroethylene, were identified from the CTD.

**Conclusion:**

The present study has effectively devised an innovative molecular subtyping methodology for patients with SSc and a diagnostic model based on machine learning to aid in clinical treatment. The study has identified potential molecular targets for therapy, thereby offering novel perspectives for the treatment and investigation of SSc.

## 1 Introduction

Systemic sclerosis (SSc), commonly referred to as scleroderma, is a rare autoimmune disease affecting connective tissues, characterized by skin and internal organ fibrosis, autoimmunity, and vasculopathy, with a significantly higher mortality rate compared to other rheumatic diseases. The progression rate, disease manifestations, and response to therapy exhibit significant variability among individuals (1, 2). Due to its low prevalence, SSc is considered an orphan disease, and its burden is substantial (3). The exact cause of SSc remains uncertain, although considerable evidence suggests that genetic and environmental factors significantly contribute to its development (4, 5). While Raynaud’s phenomenon and fatigue are common early symptoms of SSc, their presentation can vary, making it challenging for clinicians to accurately diagnose the disease (6). This diagnostic difficulty may have implications for treatment decisions and patient outcomes.

Presently, the management of SSc centers on addressing the symptoms of affected cutaneous and internal organs, including but not limited to pulmonary, renal, cardiac, pulmonary arterial hypertension, gastrointestinal, and musculoskeletal involvement (2). Conventional therapeutic approaches encompass pharmacological interventions such as cyclophosphamide (CYC) and mycophenolate mofetil (MMF), while hematopoietic stem cell transplantation (HSCT) represents a crucial treatment modality. Recent research has investigated novel pharmacological interventions for the management of SSc, such as rituximab and tocilizumab, among others. The principal immunological indicators and therapeutic objectives implicated in the pathogenesis of SSc have been identified, including IL-6, IL-4, IL-13, TGF-B, and others (7). While significant advancements have been achieved in the investigation and clinical management of SSc pathogenesis, further comprehensive inquiry remains necessary.

In the early stages of SSc, the primary event is vascular injury, which triggers endothelial activation, inflammation mediated by both innate and adaptive immune responses, vascular remodeling, and ultimately fibrosis (2, 8). As such, an examination of the gene expression profiles of peripheral blood mononuclear cells (PBMCs) in SSc patients is of particular significance in comprehending the pathogenesis, immune characteristics, subtyping, and clinical management of SSc patients. At present, SSc is typically categorized into subtypes according to the degree of skin involvement, namely diffuse cutaneous SSC and limited cutaneous SSC (9). This classification based on skin involvement holds significant clinical implications (1). Additionally, a minor subset of SSc patients, known as sine scleroderma, exhibit no skin involvement (10). Presently, there are no alternative or superior subtype definitions that can effectively guide the clinical management of SSc, which poses significant challenges in its treatment.

This study involved the collection of peripheral blood transcriptome datasets from five SSc gene expression datasets sourced from the Gene Expression Omnibus (GEO) database. Through the use of unsupervised machine learning methods, three distinct and reliable subtypes of SSc patients were identified. The exploration of the immune and molecular characteristics of these subtypes has yielded significant insights that are relevant to the advancement of research and treatment of SSc. Moreover, a machine learning diagnostic model was developed utilizing key genes to aid in the clinical management of SSc. This investigation considers the vascular alterations that occur during the initial phases of SSc and introduces an innovative and presently limited technique for characterizing SSc subtypes, providing a fresh outlook for the clinical diagnosis and management of SSc.

## 2 Materials and methods

### 2.1 Data acquisition

Peripheral blood gene expression data of SSc patients were collected from the GEO database, encompassing five datasets: GSE130953 (11), GSE22356 (12), GSE65336 (13), GSE33463 (14), and GSE179153 (15). The baseline data of the patients were extracted from the datasets. The GSE179153 dataset was utilized for constructing the machine learning diagnostic model, while the remaining datasets were employed for analysis. The analysis involved 120 SSc patient samples and 113 healthy donor samples. The microarray datasets were obtained from Affymetrix. The raw “CEL” files were acquired and subjected to background adjustment and quantile normalization to produce gene expression matrix files. The probe annotation of the expression matrix was conducted using the R ‘idmap2’ package. The correlation between patient samples within the analysis dataset was calculated utilizing the R base function ‘cor’, and samples with a correlation coefficient below 0.7 were eliminated. The batch effects between datasets were eliminated using the “ComBat” algorithm from the ‘sva’ package. Subsequently, a total of 120 samples from patients with SSc were utilized for analysis. The datasets employed in this study have been succinctly outlined in **Table 1**.

**Table 1:** The dataset used in study.

Data set	Subjects	Experiment type	Platforms	Tissue
GSE179153	49 SSc vs 25 HC	Expression profiling by array	GPL10558	Whole Blood
GSE130953	62 SSc vs 62 HC	Expression profiling by array	GPL10558	Whole Blood
GSE22356	10 SSc vs 10 HC	Expression profiling by array	GPL570	PBMC
GSE33463	19 SSc vs 41 HC	Expression profiling by array	GPL6947	PBMC
GSE65336	29SSc	Expression profiling by array	GPL570	Whole Blood

### 2.2 Immune infiltration analysis

The “xCell” package, a tool that is presently accessible for identifying cell types across various data sources (16), was utilized in our study to evaluate immune cell infiltration in SSc samples. We employed 64 cell types to characterize the peripheral blood immune cell populations of SSc patients and computed the peripheral blood immune scores.

### 2.3 Unsupervised consensus clustering in SSc

The identification of intrinsic subgroups with shared biological features can be achieved through the utilization of the “ConsensusClusterPlus” software package in R (17). In order to investigate potential subtypes of SSc patients, we employed the “ConsensusClusterPlus” package for unsupervised clustering. The K-Means algorithm based on Euclidean distance and Ward-D linkage was utilized in the analysis, with 1000 iterations performed to ensure classification stability. The cumulative distribution function (CDF) values and the incremental area under the CDF curve were employed as evaluation criteria for each cluster in the consensus clustering process. Subsequently, the clustering results were validated through the utilization of principal component analysis (PCA).

### 2.4 Identification of differentially expressed genes between subtypes

The Limma package was employed to discern dissimilarly expressed genes among subtypes, utilizing the false discovery rate (FDR) technique to regulate false positives. Significance was established at adjusted p-values of <= 0.05, while a fold change of >= 0.32 was deemed indicative of significant differences.

### 2.5 Characterization of SSc subtypes

The present study employed the “xCell” package to assess the enrichment of 64 cell types and calculate immune scores in the robust SSc subtypes, in order to characterize them. Additionally, SSc-related immune pathways were selected from published literature and gene set enrichment analysis (GSEA) results, utilizing gene sets derived from the KEGG and Reactome databases, to evaluate the enrichment of metabolic pathways among SSc patient subtypes. Furthermore, the Wilcoxon test was employed to evaluate the enrichment scores of distinct cell types and pathway activities across the three subtypes, where statistical significance was determined at a p-value threshold of less than 0.05.

### 2.6 Construction of machine learning diagnostic models

Feature genes were selected using the Least Absolute Shrinkage and Selection Operator (LASSO) algorithm, Support Vector Machine (SVM) algorithm, Random forest (RF) and Boruta, based on the intersection of 19 genes among the three robust subtypes. LASSO is a well-established algorithm in machine learning that is commonly employed for feature selection and data dimensionality reduction. SVM is a supervised machine learning algorithm that can effectively classify high-dimensional large data into a limited number of data points (support vectors), thereby achieving dimensionality reduction. The R packages “glmnet” and “e1071” were utilized to implement LASSO and SVM, respectively. The RF algorithm, which comprises multiple decision trees, was implemented using the R package “randomForest”. The Boruta algorithm is a feature selection method used to identify important features in a dataset that have statistical significance. It is implemented using the R package “Boruta”. Following the acquisition of feature genes, an assessment of the correlation between these genes and immune cells was conducted. Subsequently, the dataset was partitioned into training and validation sets in a 7:3 ratio. Diagnostic models were constructed using the SVM, RF, and Logistic Regression (LR) algorithms. The LR model, which is a generalized linear regression analysis model, is frequently employed in data mining and disease diagnosis. Its implementation is carried out through the utilization of the base function “glm” in the R programming language. Ultimately, the efficacy of the three models was evaluated in both the training and testing sets by means of Receiver Operating Characteristic (ROC) curves.

### 2.7 Identification of compounds associated with SSc

The Comparative Toxicogenomics Database (CTD) was utilized to conduct a search for SSc, with a subsequent filtration of compounds associated with the feature genes, as per the “Chemical-Gene Interactions” tab.

## 3 Results

### 3.1 Exploring subtypes in SSc

**Figure 1:**
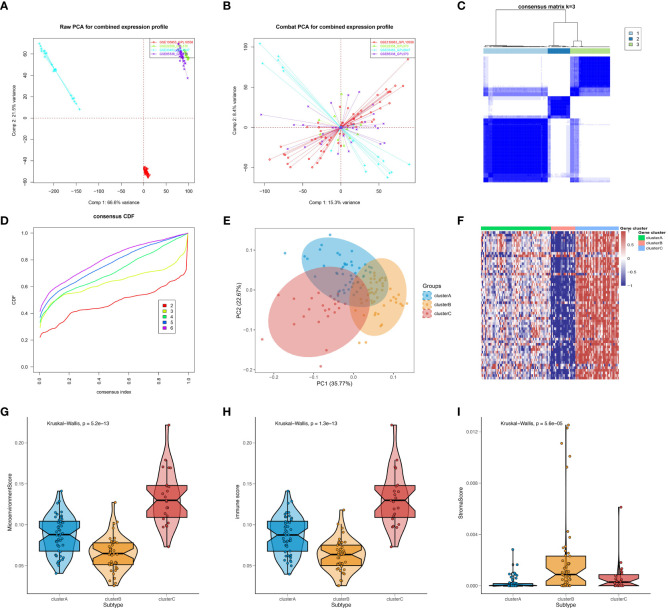
Preliminary investigation and molecular features of SSc subtypes. PCA of expression matrix for four different datasets before batch correction **(A)** and after batch correction **(B)**. **(C)** Heatmap of consensus matrix at k = 3. **(D)** Cumulative distribution frequency (CDF) curve of clustered samples. **(E)** PCA plot showing three subtypes after classification. **(F)** Heatmap showing gene expression differences between subtypes. Microenvironment scores **(G)**, immune scores **(H)**, and stromal scores **(I)** for different subtypes were calculated using the Xcell package.

The present study conducted an initial investigation into subtypes of SSc by analyzing peripheral blood expression profiles from a total of 120 SSc patients across four cohorts. To mitigate batch effects between datasets, the ComBat algorithm was employed, and the resulting batch effect-corrected changes were visualized using PCA (**Figures 1A, B**). Additionally, the xCell package was utilized to perform convolution on the peripheral blood expression profiles of the 120 SSc patients. Based on the observed differences in immune cells, the km algorithm with 1000 iterations from the ConsensusClusterPlus package was employed to perform clustering. Through an analysis of the CDF values and the incremental area under the CDF curve, we arrived at the determination that k=3 represents the optimal number of clusters, a finding that was subsequently confirmed by PCA (**Figures 1C–E**). To further investigate the differences in gene expression between the identified subtypes, we generated a heatmap, which revealed a significant upregulation of genes in cluster C, a significant downregulation of genes in cluster B, and intermediate expression levels in cluster A (**Figure 1F**). Moreover, we computed microenvironment scores, immune scores, and stromal scores for the various subtypes. The results demonstrated that cluster C had the highest microenvironment and immune scores, cluster B had the lowest scores, and cluster A fell between clusters B and C (**Figures 1G, H**), which corresponded to the heatmap results. As for the stromal score, cluster B had the highest score, cluster A had the lowest score, and Cluster C was in the middle (**Figure 1I**). Since the gene expression data were derived from peripheral blood, the stromal score might not be meaningful. However, overall, these results suggest that stratifying SSc patients based on the immune cell composition in peripheral blood is effective. In summary, the findings indicate that the stratification of SSC patients according to the composition of immune cells in the peripheral blood is a viable approach.

### 3.2 Identification of robust subtypes in SSc

**Figure 2:**
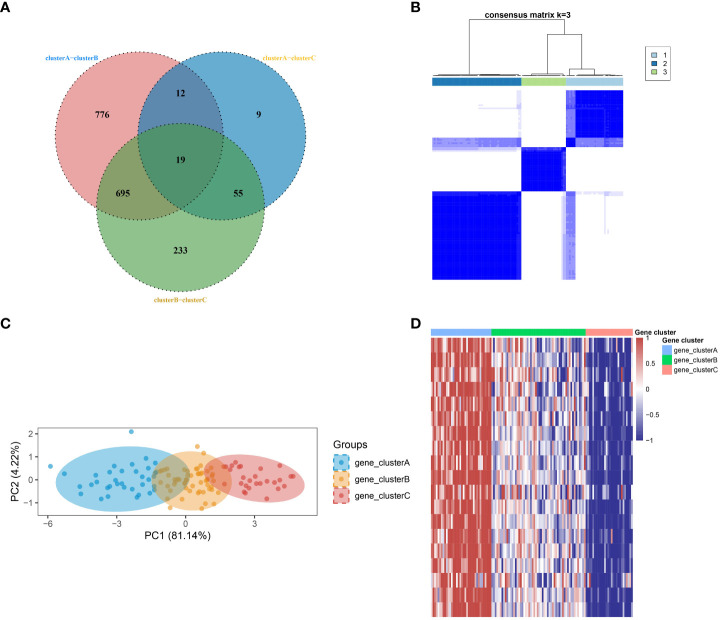
Identification of robust SSc subtypes. **(A)** Venn diagram identified 19 significant DEGs. **(B)** Three robust SSc subtypes were identified through unsupervised consensus clustering based on the 19 key DEGs. **(C)** PCA plot displays the distribution of the three subtypes. **(D)** Heatmap shows the expression differences of the 19 significant DEGs among the subtypes.

In order to develop a more comprehensive definition of SSc subtypes, the limma package was utilized to compute differential gene expression among the three subtypes. Through the implementation of a Venn diagram, 19 significant DEGs were identified (**Figure 2A**), which served as crucial factors in distinguishing the three subtypes. Subsequently, an unsupervised clustering analysis was conducted on the SSc samples, resulting in the identification of three more robust subtypes (gene_clusterA-C) using the ConsensusClusterPlus algorithm (**Figure 2B**), based on the aforementioned 19 key DEGs. The PCA further confirmed the findings (**Figure 2C**). The heatmap revealed that gene_clusterA manifested significantly elevated expression levels across all 19 genes, gene_clusterC exhibited significantly reduced expression levels, and gene_clusterB displayed moderate expression levels (**Figure 2D**).

### 3.3 Molecular features of robust subtypes in SSc

**Figure 3:**
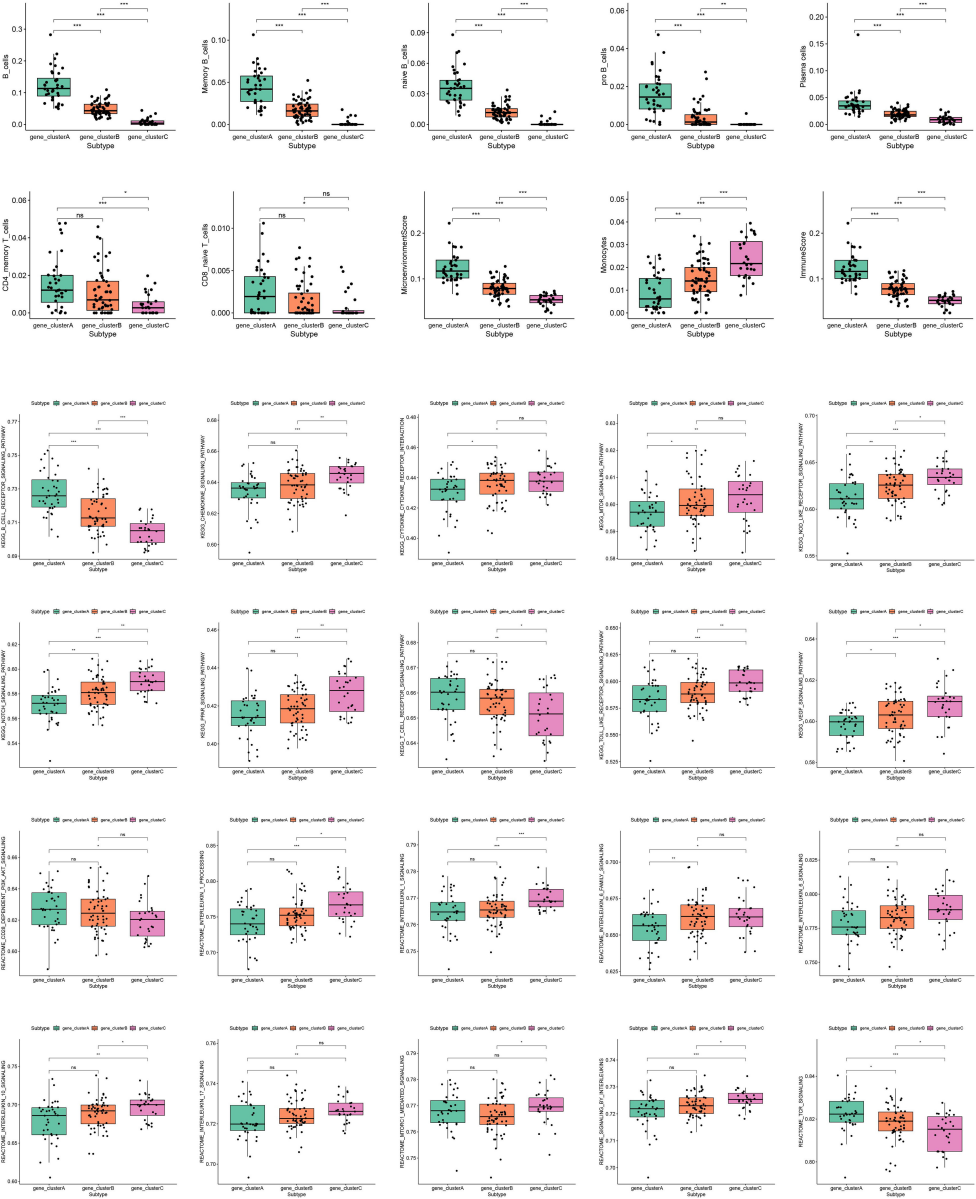
Molecular features of subtypes exhibited by immune cells and immune-related pathways. **(A)** Enrichment scores of immune cell infiltration in different subtypes. **(B)** Enrichment scores of SSc-related immune pathways in different subtypes.

In order to comprehend the molecular attributes and physiological roles of the three resilient subtypes, we conducted an investigation into their prevalence across 64 cell types and immune-related pathways (**Figure 3**). Our findings indicate that gene_clusterA subtype demonstrated a notably greater prevalence of B cells and T cells in comparison to the other subtypes, particularly in B cell-related enrichments and the B cell receptor signaling pathway. Furthermore, gene_clusterA exhibited a high degree of enrichment in TCR signaling transduction and CD28-dependent PI3K-AKT signaling. Monocytes exhibited anomalous activity in gene_clusterC, which was notably enriched in interleukin-related responses, encompassing interleukin 1, 6, 10, and 17 signalings, as well as the processing of interleukin 1. Additionally, gene_clusterC demonstrated elevated scores in various signaling pathways, including the chemokine signaling pathway, cytokine-cytokine receptor interaction, mTOR signaling pathway, Nod-like receptor signaling pathway, Notch signaling pathway, PPAR signaling pathway, Toll-like receptor signaling pathway, and VEGF signaling pathway. It is noteworthy that gene_clusterB demonstrates moderate activation throughout all cells and pathways. Utilizing these molecular characteristics, we have classified the gene_clusterA subtype as B-cell rich, gene_clusterB as intermediate, and gene_clusterC as monocyte activated.

### 3.4 Construction of machine learning diagnostic models

**Figure 4:**
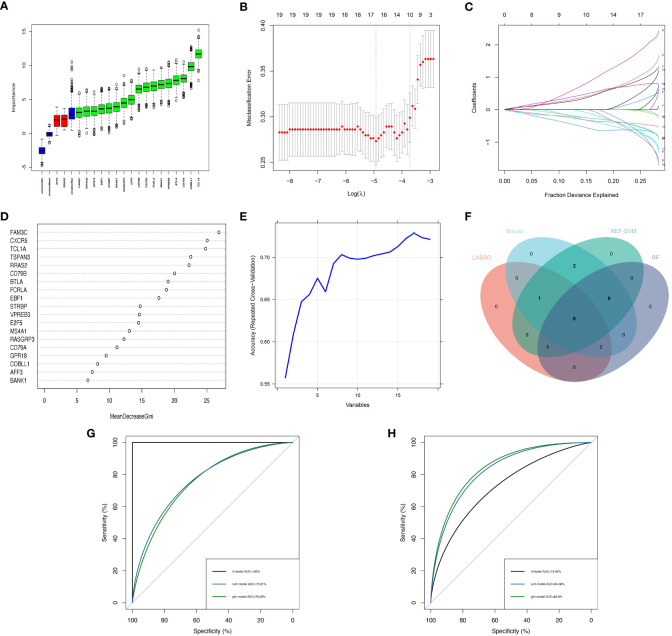
Construction of Machine Learning Diagnostic Models. **(A–E)** Feature genes screening in the Boruta, LASSO, RF and SVM algorithms. **(F)** Four machine learning algorithms selected feature genes and the Venn diagram of their intersection. AUC curves of the three models in the training set **(G)** and validation set **(H)**.

In order to enhance the precision of marker genes, we utilized SVM, LASSO regression, RF and Boruta for feature selection from a pool of 19 key DEGs (**Figures 4A–E**). Ultimately, we identified 8 feature genes, namely “FAM3C”, “BTLA”, “STRBP”, “RASGRP3”, “CD79A”, “MS4A1”, “CXCR5”, and “TCL1A” (**Figure 4F**). To establish dependable clinical classifiers for SSc subtypes, we developed classification models using SVM, RF, and LR. The dataset GSE179153 was partitioned into a training set and a validation set at a ratio of 7:3. The efficacy of the SVM, RF, and LR models was evaluated in both sets using ROC curves. In the training set, the AUC values of the SVM, RF, and LR models were 0.7591, 1.000, and 0.7609, respectively (**Figure 4G**). In the validation set, the AUC values were 0.8408, 0.7306, and 0.829, respectively (**Figure 4H**). Overall, the three machine learning models, which were based on the 8 feature genes, demonstrated exceptional predictive performance, with the SVM model exhibiting the highest performance.

### 3.5 Correlation between feature genes and immune cells

**Figure 5:**
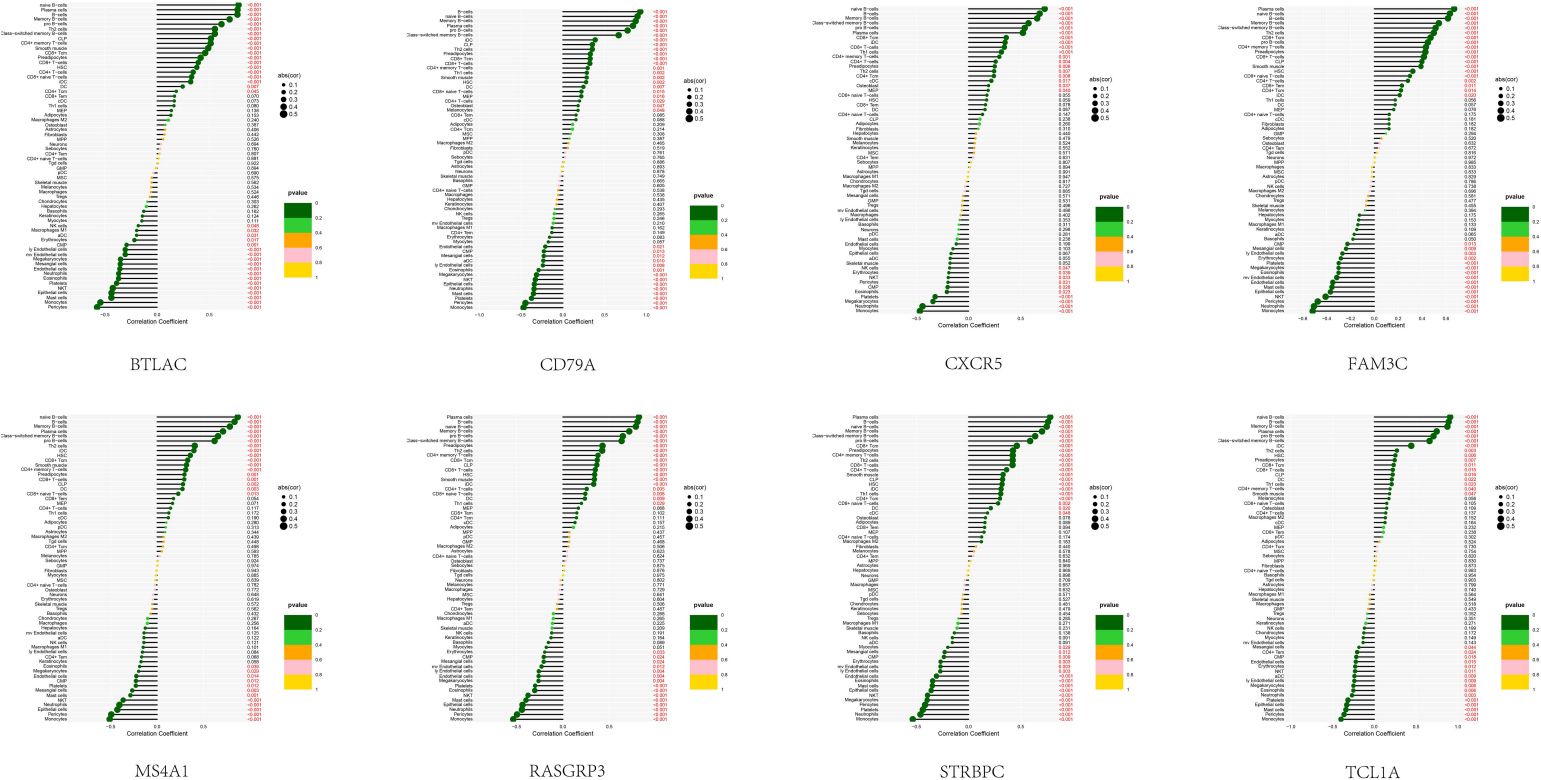
Correlation between the 8 feature genes and immune cell.

In order to examine the association between the diagnostic model and SSc, an analysis was conducted to determine the correlation between the 8 feature genes and immune cells present in the peripheral blood of SSc patients (**Figure 5**). Notably, these 8 genes exhibited a robust correlation with B cells (including naïve B cells, memory B cells, and other B cell subtypes), monocytes, and epithelial cells. This discovery is in strong agreement with our prior analysis and serves to reinforce the precision of our SSc subtype classification.

### 3.6 Exploration of potential therapeutic drugs for SSc

Nine compounds of relevance were screened in the CTD, based on the 8 feature genes (**Table 2**). The expression of BTLA is decreased by trichloroethylene, while methotrexate affects the expression of CD79A and formaldehyde results in a decrease in CD79A expression. CXCR5 expression is increased by silicon dioxide. Additionally, the sensitivity of SSc patients to paclitaxel is influenced by the FAM3C protein. Silicon dioxide has been observed to elicit an upregulation of MS4A1 expression, whereas the administration of [hydroxychloroquine + methotrexate + sulfasalazine] combination therapy has been shown to induce a downregulation of MS4A1 expression. Conversely, formaldehyde exposure has been associated with a reduction in MS4A1 expression. Analogues of silicon dioxide or [rheumatoid arthritis drugs combined with methotrexate] have both been found to result in a decrease in RASGRP3 expression. Resveratrol and benzene exposure have been linked to a decrease in STRBP expression, while trichloroethylene and tetrachloroethylene exposure have been associated with an increase in STRBP gene expression. Additional investigation is necessary to examine the correlation between said compounds and SSc.

**Table 2:** Interactions between compounds and genes related to SSc in the CTD database.

Chemical Name	Gene Symbol	Interaction
Trichloroethylene	BTLA	Trichloroethylene results in decreased expression of BTLA mRNA
Methotrexate	CD79A	Methotrexate affects the expression of CD79A mRNA
Trichloroethylene	CD79A	Trichloroethylene results in increased expression of CD79A mRNA
Formaldehyde	CD79A	Formaldehyde results in decreased expression of CD79A mRNA
Silicon Dioxide	CXCR5	Silicon Dioxide results in increased expression of CXCR5 mRNA
Paclitaxel	FAM3C	FAM3C protein affects the susceptibility to Paclitaxel
Silicon Dioxide	MS4A1	Silicon Dioxide results in increased expression of MS4A1 mRNA
Methotrexate	MS4A1	[Hydroxychloroquine co-treated with Methotrexate co-treated with Sulfasalazine] results in decreased expression of MS4A1 mRNA
Formaldehyde	MS4A1	Formaldehyde results in decreased expression of MS4A1 mRNA
Silicon Dioxide	RASGRP3	Silicon Dioxide analog results in decreased expression of RASGRP3 mRNA
Antirheumatic	RASGRP3	[Antirheumatic Agents co-treated with Methotrexate] results in decreased expression of RASGRP3 mRNA
Resveratrol	STRBP	Resveratrol results in decreased expression of STRBP protein
Benzene	STRBP	Benzene results in decreased expression of STRBP mRNA
Trichloroethylene	STRBP	Trichloroethylene results in increased expression of STRBP mRNA
Tetrachloroethylene	STRBP	Tetrachloroethylene results in increased expression of STRBP mRNA

## 4 Discussion

SSc is a chronic fibrotic disease that arises from autoimmune dysfunction (18). It poses a rare and formidable challenge for treatment (19, 20). Early vascular damage in the disease progression serves as a link between immune abnormalities and fibrosis, thereby triggering pathological cascades in multiple organs (21, 22). Furthermore, the early detection of SSc onset is arduous, and prior research has not established a definitive molecular subtype classification for SSc, and there is a dearth of valuable biomarkers for the disease. This study has successfully identified three distinct and resilient subtypes of SSc, specifically characterized as B-cell rich, intermediate, and monocytes activate types. Subsequently, a diagnostic model has been developed to aid in clinical management.

The xCell algorithm was utilized in this study to examine peripheral blood expression profiles from four datasets of SSc patients. Our findings revealed notable variations in immune cells among SSc patients, which were used to conduct preliminary clustering. Further differential analysis was performed between the identified clusters.By employing the 19 essential DEGs, we have successfully distinguished three resilient subtypes of SSc patients, namely gene_clusterA (characterized by B cell enrichment), gene_clusterB (intermediate in nature), and gene_clusterC (marked by monocyte activation). Significantly, our results were validated by prior investigations (23, 24). Patients with SSc who exhibited a subtype enriched with B cells demonstrated heightened activity of diverse B cell subpopulations in their peripheral blood, including memory and naïve B cells. Notably, B cells play a pivotal role in the pathogenesis of SSc by producing cytokines such as IL-6 and TGF-β (25, 26), engaging in self-activation with T cells (27), stimulating fibroblasts (28), and contributing to endothelial cell activation and injury (29, 30), among other pathways, which ultimately lead to the inflammatory and fibrotic phenotypic manifestations of SSc. Moreover, the B cell-enriched subtype of SSc patients exhibited a significant enrichment in two signaling pathways, namely the CD28-dependent PI3K-AKT signaling and TCR signaling. The activation of PI3K by the co-stimulatory receptor CD28 leads to the generation of PIP3 on the plasma membrane. Akt is involved in the CD28-mediated co-stimulation of T cell activation (31, 32). Furthermore, individuals with SSc who exhibit a monocyte-activated subtype demonstrate a noteworthy increase in the abundance of monocytes in their peripheral blood. This observation is consistent with prior research, such as the work of Alain Lescoat et al, which posits that monocyte adhesion may escalate in SSc due to the loss of CD52 (33). Macrophages derived from monocytes expressing CD163 or CD204 may serve as potential regulators of fibrosis in the skin of individuals with SSc (34, 35). The utilization of flow cytometry by Laure Ricard et al. revealed a noteworthy elevation in 6-Sulfo LacNAc monocytes, intermediate monocytes, and non-classical monocytes in individuals with SSc (24), with a more pronounced increase in SlanMo cells observed in those with diffuse SSc. Furthermore, the subtype activated by monocytes exhibited a notable enrichment in interleukin-mediated responses, encompassing signaling pathways for interleukin-1, interleukin-6, interleukin-10, interleukin-17, and interleukin-1 processing. Interleukins are recognized as significant contributors to the advancement of SSc (36–39). In conclusion, our identification and description of SSc subtypes may serve as promising avenues for future therapeutic research in SSc.

Dimensionality reduction was performed on the 19 key DEGs using SVM, RF, Boruta and LASSO regression, resulting in the identification of 8 feature genes, namely FAM3C, BTLA, STRBP, RASGRP3, CD79A, MS4A1, CXCR5, and TCL1A. Subsequently, clinical diagnostic models were constructed based on the aforementioned 8 feature genes. The model shows good predictive performance in both training set and validation set. BTLA, a constituent of the CD28 superfamily, plays a pivotal role as a co-signaling molecule. Its principal role involves hindering the activation and proliferation of T cells, B cells, and DC cells. Recent investigations have shed light on the notable importance of BTLA in the realm of autoimmune diseases, as it has demonstrated efficacy in mitigating conditions such as multiple sclerosis (MS), active systemic lupus erythematosus, and rheumatoid arthritis (RA) (40). STRBP is a protein that exhibits affinity for nuclear RNA in spermatids. Trang T Le et al. employed machine learning algorithms to identify a potential association between STRBP and the differentiation cluster cell surface biomarker in the blood of patients with SLE (41). CD79, consisting of CD79A and CD79B, is predominantly expressed in B cells and B-cell tumors, and plays a crucial role in the expression and function of B-cell antigen receptors. CD79A can be utilized as a primary diagnostic marker for B-cell-related diseases (42), and has been implicated in various pathological conditions. Notably, Ian R Hardy and colleagues have proposed the use of monoclonal antibodies targeting CD79B as a means to collectively suppress B cells and prevent autoimmunity, with the added benefit of facilitating rapid immune recovery, unlike other approaches that induce B cell death (43). This implies that targeting CD79A has significant potential for the treatment of SSc. The gene MS4A1, also known as CD20, encodes a surface molecule present on B-cells that plays a crucial role in their development and differentiation into plasma cells. It is worth noting that rituximab, a chimeric antibody specifically targeting CD20, has exhibited effectiveness in treating fibrotic lesions in SSc and has been approved for the management of SSc and SSc-ILD in certain countries (44). The CXCR5 receptor interacts with CXCL13, a chemoattractant that attracts B-cells. The CXCL13-CXCR5 axis fulfills various biological functions, including the regulation of cancer cell growth, proliferation, invasion, and metastasis (45). Additionally, this axis is implicated in the pathogenesis of several autoimmune diseases (46). TCL1A functions as a co-activator of the serine/threonine kinase Akt, facilitating cell survival, growth, and proliferation through various interactions. TCL1A has the ability to modulate B-cell differentiation and regulation (47). In summary, these feature genes are highly associated with immunity, which provides an important reference for exploring their role in SSc.

By utilizing the CTD, we have successfully identified 9 compounds that exhibit an association with SSc and exert an impact on the eight feature genes. Notably, methotrexate stands out as the most frequently employed immunosuppressant in SSc patients (48). The European League Against Rheumatism advocates for methotrexate as the primary treatment option for early diffuse SSc’s skin manifestations (49). The impact of Resveratrol on SSc has been noted, as it has the potential to enhance fibrosis and mitigate inflammatory responses in SSc through the modulation of the SIRT1/mTOR signaling pathway (50). Paclitaxel is a highly efficacious natural anticancer agent; however, there have been documented cases of SSc development in cancer patients undergoing paclitaxel treatment (51). The precise mechanisms underlying this phenomenon remain to be elucidated. Additionally, the other compounds, namely trichloroethylene, formaldehyde, benzene, and tetrachloroethylene, are all recognized environmental exposure factors associated with SSc. These compounds are typical occupational exposure substances, and their association with SSc has been reported in the scientific literature (52–54).

## 5 Conclusion

This study utilized peripheral blood expression profiling data to identify three molecular subtypes of SSc and examined their molecular characteristics. Additionally, a machine learning diagnostic model was developed to aid in clinical identification. Furthermore, this investigation revealed previously unexplored therapeutic targets and compounds for SSc, offering novel insights for future research in this field. Despite the utilization of rigorous bioinformatics methods, this study is not without limitations. It is imperative to conduct molecular experimental validation to corroborate the findings. Furthermore, additional comprehensive research is required to fully explore the potential therapeutic targets and related drugs for SSc.

## Data availability statement

The datasets supporting the conclusions of this article are available in the GEO repository, (https://www.ncbi.nlm.nih.gov/geo/). The accession number(s) can be found in the article.

## Ethics statement

Ethical review and approval was not required for the study on human participants in accordance with the local legislation and institutional requirements. Written informed consent from the patients/participants or patients/participants legal guardian/next of kin was not required to participate in this study in accordance with the national legislation and the institutional requirements.

## Author contributions

QW: Methodology, Project administration, Software, Supervision, Writing – original draft, Writing – review & editing. C-LL: Writing – original draft, Writing – review & editing. LW: Writing – review & editing. J-YH: Writing – review & editing. QY: Writing – review & editing. S-XZ: Writing – review & editing. P-FH: Writing – review & editing.
